# Allegheny County Medical Society

**Published:** 1889-05

**Authors:** 


### SOCIETY REPORTS.

### ALLEGHENY COUNTY MEDICAL SOCIETY.

*Special Meeting, March 19.*

DR. W. F. KNOX, M.D., PRESIDENT, IN THE CHAIR.

LAPAROTOMY FOR EXTRA-UTERINE PREGNANCY.

DR. X. O. WEBER: At the November meeting of this Society I reported a case of extra-uterine pregnancy in which I had performed abdominal section with a successful result. To-day I present the specimen of my second case of tubal pregnancy, removed by laparotomy, on February 14th, of this year.

The history of this case is, briefly, as follows:

Mrs. M., 27 years of age, married, two children, the youngest 16 months old, has been suffering with periodical attacks of severe abdominal pains for almost a year, for which she several times required medical treatment. During the five or six weeks preceding the operation, these attacks increased in frequency and severity, making her unfit to do her ordinary household duties. Walking almost always produced a great deal of suffering. On January 26

of this year I was consulted for one of these attacks of pain, which was referred to the pelvic region, principally the left side. Making a vaginal examination, I found the uterus enlarged to the size almost of a two months' pregnancy and to the left of this, in the region of the left tube, a soft, extremely tender mass, which was slightly movable. A careful bimanual examination could not be made on account of the very great sensitiveness of these parts. She had menstruated regularly every four weeks during the last eight or nine months, and was at this time still nursing her baby. At the two subsequent examinations I found no change in her condition, except, perhaps, that this tumor was slightly larger than before. The diagnosis was not quite clear, but I was inclined to the opinion that it was either a hydrosalpinx, or a pyosalpinx, more probably the latter. As her suffering at times had almost become unbearable, I advised laparotomy, to which she readily consented, but the operation was deferred until after her next menstrual period, which was now very close at hand. Menses lasted five days, and presented nothing unusual. In the afternoon of the 13th of February, the day preceding the operation, she came to my office in a carriage, from her home, for the purpose of going to Mercy Hospital. On examination I found her condition unchanged; the mass, however, seemed now decidedly larger. The riding on the rough country road from her home did not seem to have caused her as much suffering as expected, and she was cheerful, and feeling better than for several days previously. But on her way to the hospital the pains returned in unusual severity, and she arrived there faint and nearly collapsed. Several hypodermic injections of morphia made her more comfortable, but she continued very sick and sore all night. On my morning visit before the operation, she looked very pale, and was very feeble, still suffering considerable pain. Vaginal examination was not made.

On opening the peritoneal cavity dark blood escaped from the wound, and the abdomen was found containing a considerable quantity of blood, liquid and coagulated. In reaching down for the sac, on the left of the uterus, I felt a small rent in it, probably one-half inch long, which, however, in trying to bring it to the surface, was enlarged, so that all its contents escaped into the abdominal cavity. The bleeding was now very free, the blood being bright red, and easily distinguishable from the old dark blood already contained in the abdomen. The sac was now tied off, and the clots contained within the pelvic cavity turned out. After washing out the abdomen with hot distilled water, it was closed, leaving, however, a glass drainage tube. Blood continued to discharge from this tube for three days, when it was removed.

The patient rallied very nicely from the operation, and made an uninterrupted recovery, her temperature and pulse remaining perfectly normal after the fourth day. She left the hospital on the 21st day, and is now in good health.

Rupture of the tube must have taken place on her way to or at the hospital, probably as a consequence of the jolting of the carriage. This evidently was the cause of the faintness and slight collapse after her arrival at the hospital the evening before the operation.

Comparing the histories of the two cases operated on by me, we find the first case an almost typical one of ectopic pregnancy, and one of comparatively easy diagnosis to one at all familiar with this interesting anomaly, while the second case is as atypical as possible, in which I claim a diagnosis to have been entirely impossible, for there was not the slightest reason to even suspect a pregnancy, as the patient had been menstruating regularly, her last catamenial period terminating just a few days before the operation, and she was still nursing her baby at the time she came under observation.

This case illustrates again the great difficulties in diagnosing extra-uterine pregnancy, and I can not agree with Hanks, when he states that the diagnosis can be made in at least 95 per cent of cases. The case also demonstrates that this interesting affection is by no means such a very rare condition as some seem to suppose, as this is the second case occurring in my own practice within the period of four months. That this was a case of tubal pregnancy there could be very little doubt, but in order to be perfectly certain, I sent the specimen to Dr. Wm. H. Welch, of Johns Hopkins University, Baltimore, for examination, and he verified the diagnosis. The specimen, he states, consists of an ovary, part of a Fallopian tube, the intervening broad ligament, the fœtal membranes and a placenta with umbilical cord.

Dr. Duff: Two months ago I reported a case of rheumatism, or rather a case of rheumatism associated with eruptions around the joints; at the time I did not understand the case, and could not say what the outcome would be. The rash was first papillary, then vesicular, following up in the order of the joints attacked. A few days after, I found several large blebs over the shoulder, just such as we have arise after the application of cantharides plaster. As they dried up, the submaxillary glands and cervical glands began to enlarge and continue until suppuration occurred, and discharged large amounts of pus. After suppuration occurred, the young lady improved rapidly. I am still at a loss to account for the condition, and promised that I would give the result of the case.

Dr. Painter reported a case of

### CONGENITAL MALFORMATION OF THE SOFT PALATE.

Mrs. F., aged 40, a widow eight years, consulted me on account of hoarseness following a cold. Inspecting the pharynx, I found an unique anatomical relation existing between the upper part of the pharynx and the soft palate, of which the patient was ignorant. The free border of the soft palate and the palato-pharyngeus muscle on either side are carried backward and attached to the posterior wall of the pharynx, forming a diaphragm between the superior and middle divisions of the pharynx. In this dividing membrane there are two somewhat circular openings —one one-half inch, and the other one-eighth inch in diameter. These openings are in the median line. The uvula can not be distinguished. The patient can give no reason for this marked departure from the normal construction, and was ignorant of any irregularity till I asked her to permit a demonstration of her throat to this Society. She supports a family of five by washing. She frequently has a cold in the head, but experiences no difficulty in clearing the nose. She has never had noises in the ears and hears well. The sense of smell and taste is unimpaired, and her voice, save an occasional hoarseness, has never changed. The voice might be described as muffled. Her sleep is undisturbed. At least two of her children have throats normal in construction. She has had typhoid fever, and believes she had diphtheria when a child. As I demonstrate the case, it will be observed that she is well developed generally and in good health. In the absence of any ulcerative process, I conclude the case to be one of congenital malformation. The case has two interesting points: First, this malformation is uncommon; and secondly, the absence of symptoms such as one would think should follow such abnormality.

Dr. Huselton reported a case of

### COMPOUND PUNCTURED FRACTURE OF THE SKULL, produced by the calk of a horse's shoe. John T., aged 38, a farmer, was brought into the Allegheny General Hospital on the evening of January 26th, with a history of "fractured skull." He was conscious, talked rationally, pupils equal, no paralysis, and a full, slow pulse. The history, as given by himself, is as follows: He was riding in a "buckboard," leading a spirited horse by means of an ordinary halter. The horse, becoming frightened at a passing railway train, jumped upon the "buckboard," knocking the patient to the ground. He tried to rise, still holding to the strap, when the horse reared and came down, his hoof striking the patient on the head, rendering him unconscious. He did not regain consciousness for about one hour after the injury, when he walked into the hospital supported by a friend.

An examination revealed a depressed, punctured fracture of the skull, situated in the frontal bone, two inches above the right eye. The fracture was shaped like, and about the size of a large almond, and very much depressed. A sero - sanguinolent fluid, supposed to be subarachnoid, escaped from the wound, but we were unable to find an opening in the dura mater. This fluid flowed freely as long as the head was resting on the occiput, but on turning it to either side it ceased. I trephined, removing the button from the lower portion of the wound. A number of fragments, principally from the inner table, were removed and the depressed bone elevated into position. There was no hemorrhage from the interior. The wound was flushed with a solution of bichloride of mercury (1:4000). A few strands of silk were placed in the opening and brought out at the lower portion of the wound for drainage. The edges were brought together by silk sutures, and the operation completed by an antiseptic dressing. On the morning of the 27th his temperature was 100.4°, but gradually and continuously dropped to 98.4° on the 29th, and remained normal from this time on. The dressings were removed on the 30th, four days after the operation. The wound had closed by primary union, and without a drop of pus or discharge of any character. The stitches and silk for drainage were removed on this occasion, and the head was redressed, observing the same antiseptic precautions as at first. These dressings were removed on February 4th; and as every part of the original injury was healed, an ordinary nightcap bandage was applied and the patient was permitted to get up on the next day, February 5th.

The case progressed without an untoward symptom of any kind. The patient was anxious to go home on the tenth day after the operation, but was kept in the hospital as a precautionary measure until February 15th, when he was discharged cured, and the opening in the skull was apparently being rapidly closed by a bony deposit. His treatment was practically nil. The diet was liquid for the first few days. A mercurial at the outset was all he had in the way of medication.

Dr. Buchanan: I would like to say a few words on the subject of trephining. I think that Dr. Huselton had very distinct indications for his operation and it certainly was very successful. I think there are one or two points on the subject of trephining that may be dwelt on. The principal one is that the indications for trephining have entirely changed in the last few years. Formerly, there was a very great difference made between simple and compound fractures. Compound fractures were recommended to be trephined that would not have been considered proper subjects for trephining had they been simple. The presence of a simple depressed fracture, if the depression is slight, it is impossible to make out. A case of depressed fracture occurred in my practice a week ago, in which it would have been impossible for any one to make out the depression by external examination. On the following evening, when symptoms of compression came on, I opened the scalp and found the depression, removed a button of bone, elevated it, removed a clot of blood from beneath the bone, and put on a dressing. The patient afterward had no elevation of temperature, commenced to improve immediately, and is now practically well.

The second point that I would call attention to is that the secondary results of depressed fractures are very much better appreciated now than heretofore. The deficiencies in intellect and epilepsies justify more frequent resort to the trephine and elevator in simple fractures of the skull. A case may recover and pass outside of the surgeon's sight, but still be a bad result: six months, a year, or several years after, there may be a chronic inflammation of the membranes of the brain or some damage done to the brain by plastic effusion, which will result in epilepsy or other troubles. I would therefore think that Dr. Huselton, even if there had been no compound nature in this fracture, would have been perfectly justified in elevating it, and I would go even so far as to say that when a fracture of the skull is suspected, if there is even a suspicion of depression, an exploratory operation through the scalp should be undertaken, because if there is no depression, such an operation would not hurt the patient a particle, and if there is a depression, it is exceedingly important to know it and act accordingly.

Dr. Munn: In connection with Dr. Buchanan's remarks on trephining depressed fractures, I will take the opportunity to relate a case which I met in my practice a year ago in April. A man was thrown out of a wagon by a runaway horse, and on being picked up, a depressed fracture was discovered on the upper posterior corner of the right parietal bone. He was taken to his home and there the propriety of an operation was spoken of but it was declined by the friends of the patient. He passed out of my hands, went under the care of a homœopathic physician and eventually recovered after remaining unconscious for seven days; he had had hemorrhage from the nose and the ear. Now, after the lapse of eleven months, he presents a decidedly marked depression in the region of the injury, has double vision, slight paralysis of the right arm, slight paralysis of the right leg, has some aphasia and a slight paralysis of the left side of the trunk. I think the case to-day presents every indication for operation, but the operation was not performed at the time it should have been. Since the injury, he has had two epileptic seizures, nothing of the kind ever having occurred to him before.

Dr. Huselton: I endorse everything that has been said by Dr. Buchanan and Dr. Munn. I would also add, I think we are too apt to overlook the importance of a fracture of the skull; under modern antiseptic treatment, I think trephining a comparatively safe operation and in every case I think that where there is reason to suspect a depressed fracture of the skull, the trephine is a proper precautionary measure to be resorted to.

Dr. W. P. Munn presented a specimen, obtained from a cadaver of unknown history, of

ENTIRE ABSENCE OF THE INNOMINATE ARTERY.

At its place of origin the two common carotids arise together, then the left subclavian is given off, and last arises the right subclavian, which passes towards the right, behind the other three vessels.

Dr. Buchanan: I have a former patient present whom I wish to exhibit. His case I reported to the Society three or four months since. He is a man whose patella I wired. I have not before been able to present him to the Society; I wished to present him at that time, but, as I explained, he got out of my reach. I met him on the street some time ago and found that he had a very good result, and I thought I would show him to the Society. He was away from his laboring work just eight weeks.

(Patient exhibited.) You will observe that there is no separation whatever to be discovered between the fragments, and the joint movements are perfect. The limb is, to all appearances, as good as its mate.

Dr. Murdoch: Dr. Buchanan is to be congratulated on the result of this case. So far as can be told by an examination of this man's leg, the union is perfect; there seems to be a bony union between the fragments. I say seems to be, because I do not believe it is so; I very much doubt if bony union ever takes place in a fracture of the patella, owing to the fact that many specimens have been thought to be bony, but when examined after death and the bones subjected to a process of boiling, it has been found the union was only fibrous after all. But if it is fibrous, it is just as good as if it were bony and just as useful, because there is no separation of the fragments, and by scarcely any other treatment could the two fragments be brought into such close apposition; but anybody who knows the difficulty of treating fracture of the patella knows how difficult it is to keep them in apposition, and that if they are not kept so, the patient is maimed for life. The only objection to this operation that can be raised is the danger of it, but under antiseptic precautions, where they are thoroughly carried out, it is probable that the danger will be but little, but it is a melancholy fact that, notwithstanding the perfection to which antiseptic dressings and surgery have been brought, this operation, even in the hands of the best surgeons, is frequently disastrous, that is the cutting down on the knee joint, freshening the edges of the bony surface and wiring them together. When I was in New York a year ago last spring, Dr. Sands told me of two cases that he had known where the patients had suffered amputation and had eventually died, where this operation was attempted, and only about a month ago, Dr. Stimson, at a meeting of the Academy of Medicine, in New York, stated that, during the past summer he had known three cases where an operation had been done in New York and the patients had in all three cases suffered amputation afterward, so that even in the hands of the best surgeons, and with the greatest care taken, it is a dangerous operation, and surgeons have been endeavoring to find one that is less dangerous, that will accomplish the desired result; whether they will succeed or not remains to be seen. About three or four weeks ago, Dr. Stimson, after making the remarks I have stated, exhibited five cases where he had tied the patella together subcutaneously, and the procedure seemed to me so simple and so likely to be successful that I think it should be tried, and if it succeeds, it will be much simpler than this operation and, I believe, safer. The operation is so simple that I will just show it here on the blackboard, if I can. (Drawing made by the doctor on the blackboard, exhibiting the method of operating.) I tried this last Saturday on an old lady, sixty years of age. I am not able to do what Dr. Buchanan has done, bring my patient here, and perhaps I never shall be able to do so. The patient is perfectly comfortable, and so far as anybody can tell, after this short treatment, bids fair to have a good result. I do not bring this up to criticise Dr. Buchanan. I am very glad to have had an opportunity to see Dr. Buchanan's case, the first one, I believe, that has been operated on in our country.

Dr. Huselton: I want to congratulate Dr. Buchanan on the successful issue of his case. I had the pleasure of being present when he operated, and am glad to say that I think the operation was very carefully and skilfully performed. At the same time, I do not believe the operation will ever become a popular one; I think that opening so large a joint as the knee-joint is too hazardous, and attended with too much danger, particularly when we are having very good results by the old method. I have had several cases, at least three or four, in my practice, the last one occurring about two years ago, treated by the old method, and the result everything that could be desired. I did claim the union was bony; however, I think this is not the case, but if ligamentous or fibrous, it is almost impossible to detect the fact. I exhibited the case to at least one person here and would be glad to present the case to the society at some future time for their inspection.

Dr. Buchanan: I am very glad Dr. Murdoch presented this new method of treatment. I considered that method shortly after I had done this operation. It was then first brought to my notice. It occurred to me that this certainly is a much safer operation than the open method, but it is open to two theoretical objections; whether they are real objections, time alone will tell. The first is that it will probably in a great many cases, if not the majority, be impossible to approximate the fragments exactly by this method. I should think the anterior borders of the patella, by this method, would be tilted a little backward, and that would keep the surfaces from coming together. In wiring a bone, it is sometimes a difficult matter to get the surfaces exactly apposed, even when you have everything open before you and are able to handle the parts, and of course it is very much more difficult when you are doing it subcutaneously. And the second objection that I would suppose to exist in regard to this method is, that the torn fragments of the capsule of the joint float in between the fragments. I believe it has been proposed to pass a needle in and hook these out from between the fragments. At all events, I should imagine from the case of this man, at least, that it would be very difficult to get these shreds from between the broken bones, and it is said by a number of good surgeons (Prof. Macewen was the first, I believe, to state it,) that this is a chief cause of non-union, or rather, of the failure of bony union in this fracture. In regard to Dr. Huselton's results, I think he is to be congratulated. I don't think that the result which he has mentioned is otherwise than exceptional in these cases by the non-operative methods of treatment.

